# Integrated Prognostic Stratification After Perioperative FLOT in Gastric and Gastroesophageal Junction Adenocarcinoma: A Multicenter Real-World Study

**DOI:** 10.3390/jcm15166300

**Published:** 2026-08-14

**Authors:** Seda Jeral Evinç, Zeliha Birsin, Selin Cebeci, Ahmet Başgöze, Çağla Eyüpler Akmercan, Tuğba Kaya, Burak Paçacı, Murat Sarı, İlknur Deliktaş Onur, Ayberk Bayramgil, Özgecan Dülger Kaya, Lamia Şeker Can, Hamza Abbasov, Emir Çerme, Ebru Çiçek, Süheyla Atak, Süleyman Sami Güzel, Kubilay Tay, Nebi Serkan Demirci, Özkan Alan

**Affiliations:** 1 Department of Medical Oncology, Cerrahpaşa Faculty of Medicine, Istanbul University-Cerrahpaşa, Istanbul 34098, Türkiye; zelihabirsin@gmail.com (Z.B.); sellcebeci@gmail.com (S.C.); hamzaabbasov90@gmail.com (H.A.); emircrm34@gmail.com (E.Ç.); ebruhct@gmail.com (E.Ç.); suheylakeles93@gmail.com (S.A.); suleymanguzel34@hotmail.com (S.S.G.); kubilaytay@hotmail.com (K.T.); drserkannebi@yahoo.com (N.S.D.); ozkan.alan@iuc.edu.tr (Ö.A.); 2 Department of Medical Oncology, Prof. Dr. Süleyman Yalçın City Hospital, Istanbul 34722, Türkiye; drahmetbasgoze@gmail.com (A.B.); cagla.eyupler@gmail.com (Ç.E.A.); 3 Department of Medical Oncology, Kartal Dr. Lütfi Kırdar City Hospital, Istanbul 34865, Türkiye; tugbakaya89@hotmail.com; 4 Department of Medical Oncology, Marmara University Pendik Training and Research Hospital, Istanbul 34899, Türkiye; drpacaci@gmail.com (B.P.); murat.sari@marmara.edu.tr (M.S.); 5 Department of Medical Oncology, Dr. Abdurrahman Yurtaslan Ankara Oncology Training and Research Hospital, Ankara 06200, Türkiye; ilknurdeliktas382@gmail.com; 6 Department of Medical Oncology, Ümraniye Training and Research Hospital, Istanbul 34764, Türkiye; ayberkbayramgil@gmail.com (A.B.); ozgecandr@gmail.com (Ö.D.K.); 7 Department of Medical Oncology, Bezmialem Vakıf University Faculty of Medicine, Istanbul 34093, Türkiye; lamia.seker1@gmail.com

**Keywords:** stomach neoplasms, gastroesophageal junction adenocarcinoma, perioperative chemotherapy, FLOT, prognosis, risk stratification, survival analysis, comorbidity, systemic inflammation, carcinoembryonic antigen

## Abstract

**Background/Objectives**: Outcomes after perioperative FLOT for gastric and gastroesophageal junction adenocarcinoma remain variable, even among patients treated with curative intent. Although postoperative pathological findings are central to risk assessment, they do not fully capture patient-related factors that may influence recovery, treatment completion, and survival. This multicenter study evaluated routinely available clinical, laboratory, and pathological variables and assessed whether integrating these variables could improve postoperative prognostic stratification. **Methods**: We retrospectively analyzed 173 patients with locally advanced gastric or gastroesophageal junction adenocarcinoma treated with perioperative FLOT across seven oncology centers in Türkiye. Overall survival (OS) was the primary endpoint. Baseline inflammatory/nutritional status, pretreatment carcinoembryonic antigen (CEA), postoperative nodal status, and comorbidity burden were assessed. An exploratory modified FLOT Prognostic Score (mFPS) was constructed by assigning one point each for high inflammatory/nutritional risk, elevated CEA, ypN-positive disease, and age-adjusted Charlson Comorbidity Index ≥ 5. **Results**: At a median follow-up of 39.7 months, median OS was 41.4 months, with estimated 2-year and 5-year OS rates of 69% and 44%, respectively. Surgery was performed in 164 patients, and 83 patients completed planned adjuvant chemotherapy. In multivariable analysis, ypN-positive disease, higher comorbidity burden, and high inflammatory/nutritional risk were independently associated with shorter OS. Among the 126 patients with complete data available for all score components, the mFPS stratified patients into groups with significantly different outcomes: median overall survival was not reached in the low-risk group, compared with 56.1 months in the intermediate-risk group and 18.7 months in the high-risk group. **Conclusions**: Survival after perioperative FLOT was not determined by residual disease burden alone. Integrating postoperative nodal status with baseline host-related factors may help identify patients at increased risk of adverse outcomes following curative-intent treatment. The proposed score should be considered exploratory and requires external validation before clinical application.

## 1. Introduction

Gastric cancer continues to be associated with high cancer-related mortality worldwide despite major advances in surgical and systemic treatment approaches [1,2]. For patients with locally advanced gastric and gastroesophageal junction adenocarcinoma, perioperative fluorouracil, leucovorin, oxaliplatin, and docetaxel (FLOT) chemotherapy is currently considered the standard treatment in medically fit patients after the survival advantage reported in the FLOT4 trial [3]. More recently, the addition of immunotherapy to perioperative chemotherapy has demonstrated promising efficacy in this setting, further expanding the therapeutic landscape of resectable gastric cancer [4]. However, outcomes remain variable even among patients treated with curative-intent multimodal therapy, suggesting that anatomical staging alone may not fully capture the risk profile of this population [1,5].

For this reason, identifying reliable prognostic factors after perioperative treatment remains clinically relevant. Pathologic stage following neoadjuvant treatment remains one of the strongest prognostic factors in gastric cancer [6]. In particular, residual nodal disease and poor nodal regression after perioperative chemotherapy have consistently been associated with inferior survival outcomes and may reflect reduced sensitivity to systemic treatment [7]. Becker et al. reported marked variability in histopathologic regression after neoadjuvant therapy, even among patients treated within similar clinical settings [6]. This variability matters in daily practice, because patients with similar postoperative pathological findings may still have markedly different clinical courses. Factors related to the patient, including inflammatory status, nutritional reserve, and comorbidity burden, may therefore need to be considered together with postoperative pathologic findings.

Growing evidence suggests that gastric cancer is a biologically heterogeneous disease, and patient outcomes cannot always be explained by anatomical staging alone. In addition to pathological findings, molecular characteristics, gastric and intestinal phenotypes, systemic inflammatory response, nutritional status, and host-related factors have all been associated with prognosis. These observations have increased interest in integrated prognostic approaches that combine complementary clinicopathological and host-related variables to achieve a more comprehensive assessment of postoperative risk. This evolving understanding provides the rationale for exploring prognostic models that integrate routinely available clinicopathological and host-related variables rather than relying on a single prognostic factor [8]. Although the GRIM score has subsequently been evaluated in several malignancies, its prognostic value in patients receiving perioperative chemotherapy remains less well established [9]. Therefore, its potential role in patients treated with perioperative FLOT warrants further investigation.

Serum tumor markers may also add prognostic information in this setting. Carcinoembryonic antigen (CEA) is routinely assessed before treatment in patients with gastric cancer and may reflect tumor-related biological burden. Previous studies have shown that elevated pretreatment CEA is associated with poorer survival outcomes in gastric cancer [10]. Although CEA alone is not sufficient for individual risk prediction, it may be useful when interpreted together with pathological findings and patient-related factors. Therefore, pretreatment CEA represents a simple and clinically available marker that can be incorporated into an integrated risk assessment.

Systemic inflammation and nutritional status may contribute to differences in survival among patients with gastric cancer. Markers such as the neutrophil-to-lymphocyte ratio (NLR), prognostic nutritional index (PNI), and Glasgow Prognostic Score (GPS) have been evaluated in several studies involving operable gastrointestinal malignancies [11,12]. Across these studies, an unfavorable inflammatory or nutritional profile has generally been linked to poorer oncologic outcomes [11]. More recently, nutritional deterioration during perioperative FLOT chemotherapy was associated with shorter recurrence-free survival in patients with locally advanced oesophagogastric adenocarcinoma [12]. These observations have increased interest in composite inflammation-based prognostic scores. Originally introduced in patients treated in immunotherapy trials, the Gustave Roussy Immune (GRIM) score combines serum albumin, lactate dehydrogenase (LDH), and NLR [13].

Patient-related characteristics may also contribute to differences in treatment outcomes. Comorbidities can affect postoperative recovery, tolerance to perioperative chemotherapy, and survival. The age-adjusted Comorbidity Index (aCCI) is frequently used in oncology practice to estimate comorbidity-associated mortality risk, and higher comorbidity burden has been associated with poorer survival in patients with gastric cancer [14,15]. Nevertheless, data integrating the inflammatory markers, postoperative nodal status, tumor-related factors, and comorbidity burden in patients receiving perioperative FLOT remain limited.

In daily practice, postoperative risk after FLOT is rarely explained by a single variable. We therefore examined whether routinely available markers reflecting host status, tumor burden, residual nodal disease, and comorbidity could be integrated to better describe survival risk after perioperative treatment. In the present study, we investigated the prognostic value of the GRIM score in patients with locally advanced gastric or gastroesophageal junction cancer treated with perioperative FLOT chemotherapy. We also explored a modified FLOT Prognostic Score (mFPS), integrating systemic inflammatory status, pretreatment CEA, postoperative nodal status, and comorbidity burden. The aim was not to develop a validated prediction model, but rather to explore whether these routinely available variables could be combined into a clinically interpretable framework for postoperative risk stratification.

## 2. Materials and Methods

### 2.1. Study Design and Patient Population

This multicenter retrospective cohort study included consecutive patients treated between January 2017 and April 2025 across seven oncology centers in Türkiye. The study was conducted and reported in accordance with the STROBE statement [16]. In addition, the reporting of the exploratory development of the mFPS was informed, where applicable, by the principles of the TRIPOD statement [17]. Patients with locally advanced gastric or gastroesophageal junction adenocarcinoma who were treated with perioperative FLOT chemotherapy with curative intent were retrospectively evaluated. The study was approved by the institutional ethics committee. Given the retrospective nature of the study, the requirement for written informed consent was waived.

Patients were eligible if they were 18 years or older, had histologically confirmed gastric or gastroesophageal junction adenocarcinoma, and had locally advanced non-metastatic disease at baseline. All included patients received perioperative FLOT chemotherapy with curative intent and had completed at least two cycles of neoadjuvant FLOT. Baseline clinical and laboratory data, including the variables required for calculation of the GRIM score, and follow-up information for survival analysis had to be available. Patients with distant metastatic disease at diagnosis, non-adenocarcinoma histology, prior systemic treatment for gastric cancer before FLOT, fewer than two cycles of neoadjuvant FLOT, missing essential baseline laboratory data, or insufficient follow-up information were excluded.

Patients were included regardless of whether they subsequently underwent surgery, provided that FLOT had been initiated with curative intent. This approach was chosen to reflect the real-world perioperative treatment pathway, including patients who could not proceed to surgery because of progression, death, or patient refusal after neoadjuvant treatment.

A total of 173 patients met the eligibility criteria and were included in the final analysis. Baseline variables included age, sex, Eastern Cooperative Oncology Group performance status, clinical stage, clinical T and N categories, tumor localization, histopathologic subtype, mismatch repair (MMR) status when available, pretreatment CEA and CA 19-9 levels, positron emission tomography/computed tomography (PET/CT)-derived primary tumor maximum standardized uptake value (SUVmax) when available, aCCI, NLR and GRIM score. Treatment and outcome variables included the number of neoadjuvant and adjuvant FLOT cycles, chemotherapy completion, surgical status, reasons for not undergoing surgery, postoperative pathologic findings, disease progression, death, and follow-up duration. In the final cohort, 164 patients underwent surgery, whereas 9 patients did not proceed to surgery because of disease progression, death, or patient refusal.

### 2.2. Treatment and Perioperative Assessment

Perioperative FLOT chemotherapy was administered according to institutional practice at each participating center. The intended treatment strategy consisted of neoadjuvant FLOT followed by curative-intent surgery and postoperative chemotherapy when clinically feasible. The number of delivered neoadjuvant and adjuvant FLOT cycles was recorded, together with treatment completion status and documented reasons for discontinuation.

Neoadjuvant chemotherapy completion was defined as receipt of the planned preoperative treatment course without premature discontinuation. Adjuvant chemotherapy was assessed separately because postoperative treatment delivery may be influenced by surgical recovery, toxicity, early progression, death, or patient preference.

### 2.3. Pathologic Evaluation

Postoperative pathologic assessment was performed locally at each participating center and was not centrally reviewed. Pathologic complete response was defined as the absence of residual viable tumor in the resected specimen. Post-treatment pathologic T and N categories were recorded as ypT and ypN stages.

For prognostic analyses, pathologic nodal status was categorized as ypN0 versus ypN-positive disease. This classification was selected because residual nodal involvement after neoadjuvant therapy is clinically meaningful and can be applied consistently across centers. Patients who did not undergo surgery, or those with unavailable postoperative pathologic staging, were not included in analyses requiring ypT or ypN status, but they remained in the overall survival (OS) cohort when survival data were available.

### 2.4. GRIM Score, Comorbidity, and Laboratory Variables

The GRIM score was calculated before treatment initiation using serum albumin, LDH, and NLR as originally described by Bigot et al. [13]. One point was assigned for each adverse factor: serum albumin < 35 g/L, LDH above the institutional upper limit of normal, and NLR > 6. The total score ranged from 0 to 3. Patients were categorized as having a low GRIM score (0–1 points) or high GRIM score (2–3 points). In this cohort, 157 patients were classified as having a low GRIM score and 16 as having a high GRIM score.

Comorbidity burden was assessed using the age-adjusted Charlson Comorbidity Index (aCCI) [18,19]. In accordance with the original validated methodology, one additional point was assigned for each decade over 50 years, while localized solid tumors contributed 2 points. The conventional aCCI scoring system was applied without modification. For survival analyses, patients were categorized using an aCCI cutoff of ≥ 5. This threshold was selected with reference to previous studies in gastric cancer and other solid malignancies demonstrating the prognostic value of the aCCI, rather than being derived from the present dataset, and was also consistent with the distribution of aCCI values observed in the present cohort [15,20,21,22,23]. Pretreatment CEA and CA 19-9 levels were obtained from baseline laboratory measurements. CEA was analyzed using a cutoff of 5 ng/mL, based on the conventional upper limit of normal used in routine clinical practice. PET-derived primary tumor SUVmax was recorded when available but was not used as a component of the prognostic score.

### 2.5. Development of the Modified FLOT Prognostic Score

The modified FLOT Prognostic Score was developed to combine clinically accessible variables reflecting different prognostic domains after perioperative treatment. Candidate variables were selected a priori to represent four clinically distinct prognostic domains: systemic inflammation, tumor-related biomarker burden, residual nodal disease, and comorbidity burden. The number of variables included in the final score was intentionally limited to reduce model overfitting. The score included four variables: GRIM score, pretreatment CEA level, postoperative nodal status, and aCCI.

Each adverse factor was assigned one point: a high GRIM score, elevated CEA, ypN-positive disease, and aCCI ≥ 5. The total score therefore ranged from 0 to 4. Of the 173 patients included in the overall cohort, 126 had complete data for all four score components and were therefore eligible for mFPS calculation. Patients were stratified into three groups according to the cumulative score: low risk (0 points), intermediate risk (1–2 points), and high risk (3–4 points). This grouping was chosen to provide a practical risk classification that could be interpreted without a complex formula and applied in routine clinical practice. The mFPS was designed as an exploratory and clinically interpretable risk classification rather than a statistically optimized prediction model. Its four components were selected a priori to represent distinct prognostic domains and were therefore assigned equal weight. We did not derive component-specific weights from the regression coefficients observed in the present cohort because these estimates were obtained from a relatively small complete-case dataset and were not internally or externally validated. Deriving weighted coefficients under these circumstances would have produced a score that was more closely tailored to the derivation cohort and therefore less suitable for use without independent validation. Equal weighting was therefore adopted to preserve clinical interpretability, maintain methodological simplicity, and minimize the risk of overfitting. Accordingly, the proposed score should be regarded as an exploratory framework that requires appropriate internal and external validation before clinical application. The components and risk categories of the mFPS are summarized in Supplementary Table S1.

### 2.6. Outcomes

The primary endpoint was OS. Overall survival was defined as the time from diagnosis to death from any cause or last follow-up. Patients who were alive at the last follow-up were censored on that date.

Secondary endpoints included survival according to GRIM score and mFPS risk groups, treatment completion, surgery rate, pathologic complete response, disease progression, and mortality.

### 2.7. Statistical Analysis

Continuous variables were summarized as the median and interquartile range. Categorical variables were reported as numbers and percentages. Group comparisons were performed using the chi-square test or Fisher’s exact test for categorical variables and the Mann–Whitney U test or Kruskal–Wallis test for continuous variables, as appropriate. Missing data were not imputed. Analyses were performed using available cases for each variable, and the number of patients included in each analysis was reported where applicable. For analyses involving the mFPS, only patients with complete data for all score components were included. Variables with substantial missingness, such as mismatch repair status, were summarized descriptively and were not included in multivariable survival models.

OS was estimated using the Kaplan–Meier method, and survival curves were compared with the log-rank test. Univariable Cox proportional hazards regression analyses were performed to identify factors associated with OS. Variables with clinical relevance and variables associated with survival in univariable analysis were considered for multivariable Cox regression. Hazard ratios were reported with 95% confidence intervals.

The prognostic performance of the mFPS was assessed by examining survival separation across predefined risk groups. Because no independent validation cohort was available, the prognostic score was considered exploratory, and model performance was assessed within the derivation cohort. A two-sided *p* value < 0.05 was considered statistically significant. Statistical analyses were performed using IBM SPSS Statistics, version 25.0 (IBM Corp., Armonk, NY, USA).

## 3. Results

### 3.1. Patient Characteristics

A total of 173 patients with gastric or gastroesophageal junction adenocarcinoma treated with perioperative FLOT between January 2017 and April 2025 were included. The median age was 61 years, and 57 patients were aged 65 years or older. The cohort was predominantly male. Baseline functional status was generally preserved, with most patients having an Eastern Cooperative Oncology Group performance status (ECOG PS) of 0 or 1; only six patients had an ECOG PS of 2. Clinical stage III disease was the largest subgroup, and most patients had either T3–T4 tumors or clinically node-positive disease at baseline. Primary tumor location was distributed mainly across the cardia, corpus/fundus, and antrum, while gastroesophageal junction tumors accounted for a smaller proportion of cases. Adenocarcinoma was the most common histologic subtype. Signet ring cell carcinoma was present in nearly one-quarter of the cohort. MMR status was available for 99 patients; among these, 90 tumors were proficient MMR and 9 were deficient MMR. Median baseline CEA and carbohydrate antigen 19-9 (CA 19-9) levels were 2.81 ng/mL and 17 U/mL, respectively. The median baseline primary tumor SUVmax on PET/CT was 10.6. aCCI was available for 136 patients, with a median value of 4.

Baseline demographic, clinical, pathological, and laboratory characteristics of the study cohort are summarized in Table 1.

### 3.2. Treatment Delivery and Pathological Outcomes

The median number of neoadjuvant FLOT cycles was 4, and 142 (82%) patients completed planned neoadjuvant chemotherapy. Surgery was performed in 164 (94.7%) patients. Among the nine patients who did not undergo surgery, the reasons were disease progression in six patients, death in one patient, and refusal in two patients. Pathological complete response was observed in seven patients (4.0%). Residual ypT3–T4 disease was observed in 107 patients with available pathological staging. Pathological nodal involvement was also common after neoadjuvant treatment, with ypN1–3 disease observed in 94 patients. Adjuvant treatment was initiated for 133 patients. Although the median number of adjuvant FLOT cycles was 4, planned adjuvant chemotherapy was completed in 83 patients. The most frequent reason for non-completion was treatment-related toxicity, followed by patient refusal, disease progression, death, and other causes.

Treatment delivery, surgical outcomes, pathological findings, and follow-up events are shown in Table 2.

### 3.3. Survival Outcomes

**Figure 1:**
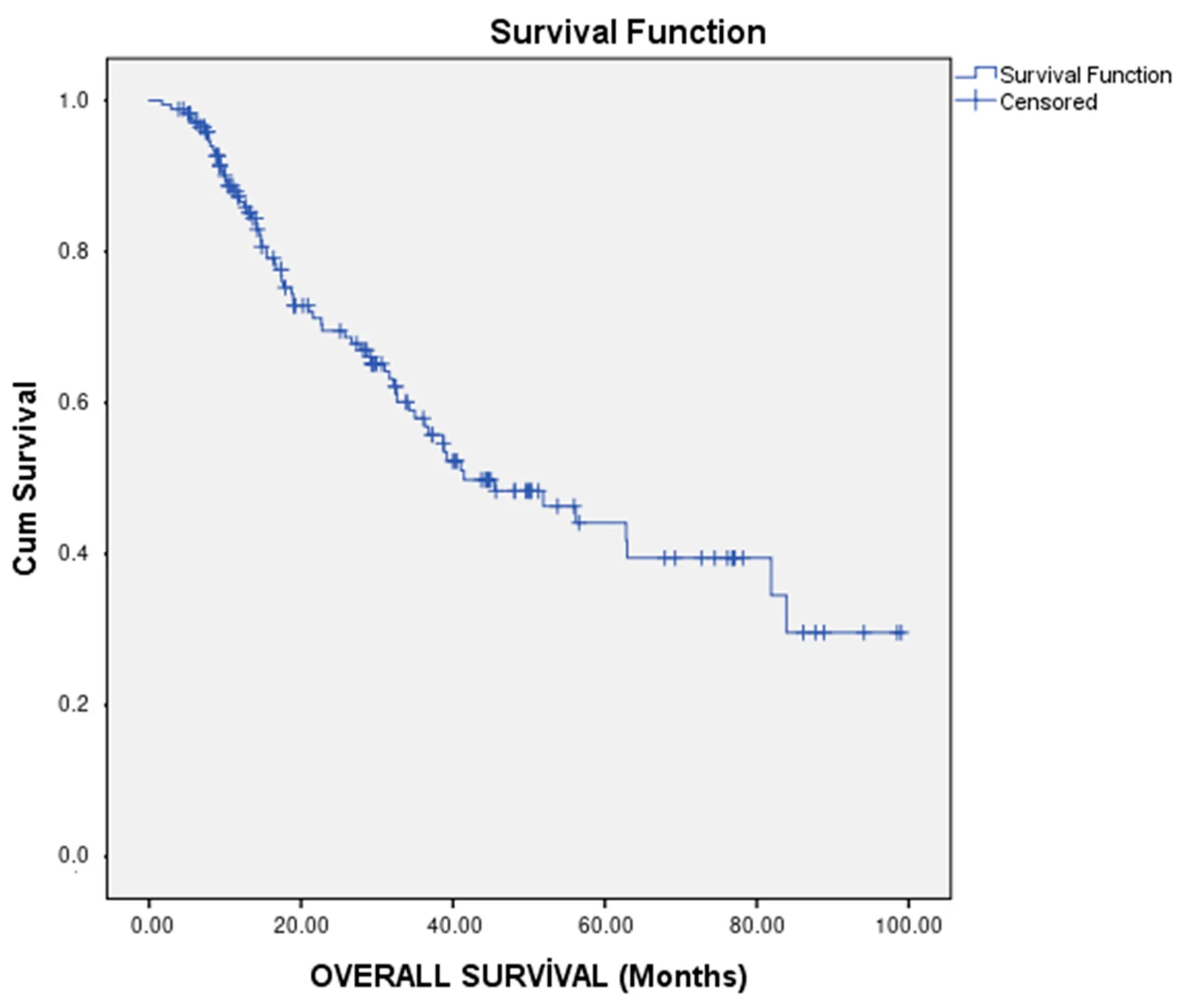
Kaplan–Meier curve for OS in the entire cohort.

At a median follow-up of 39.7 months, 61 patients had experienced disease progression and 69 patients had died. Median OS in the entire cohort was 41.4 months. The estimated 2-year and 5-year OS rates were 69% and 44%, respectively. The Kaplan–Meier curve for OS in the entire cohort is shown in Figure 1.

Kaplan–Meier curve showing OS among 173 patients with gastric or gastroesophageal junction adenocarcinoma treated with perioperative FLOT-based chemotherapy. Median OS was 41.4 months, and the estimated 2-year and 5-year OS rates were 69% and 44%, respectively. Censored observations are indicated by tick marks.

FLOT, fluorouracil, leucovorin, oxaliplatin, and docetaxel; OS, overall survival.

### 3.4. GRIM Score and Overall Survival

**Figure 2:**
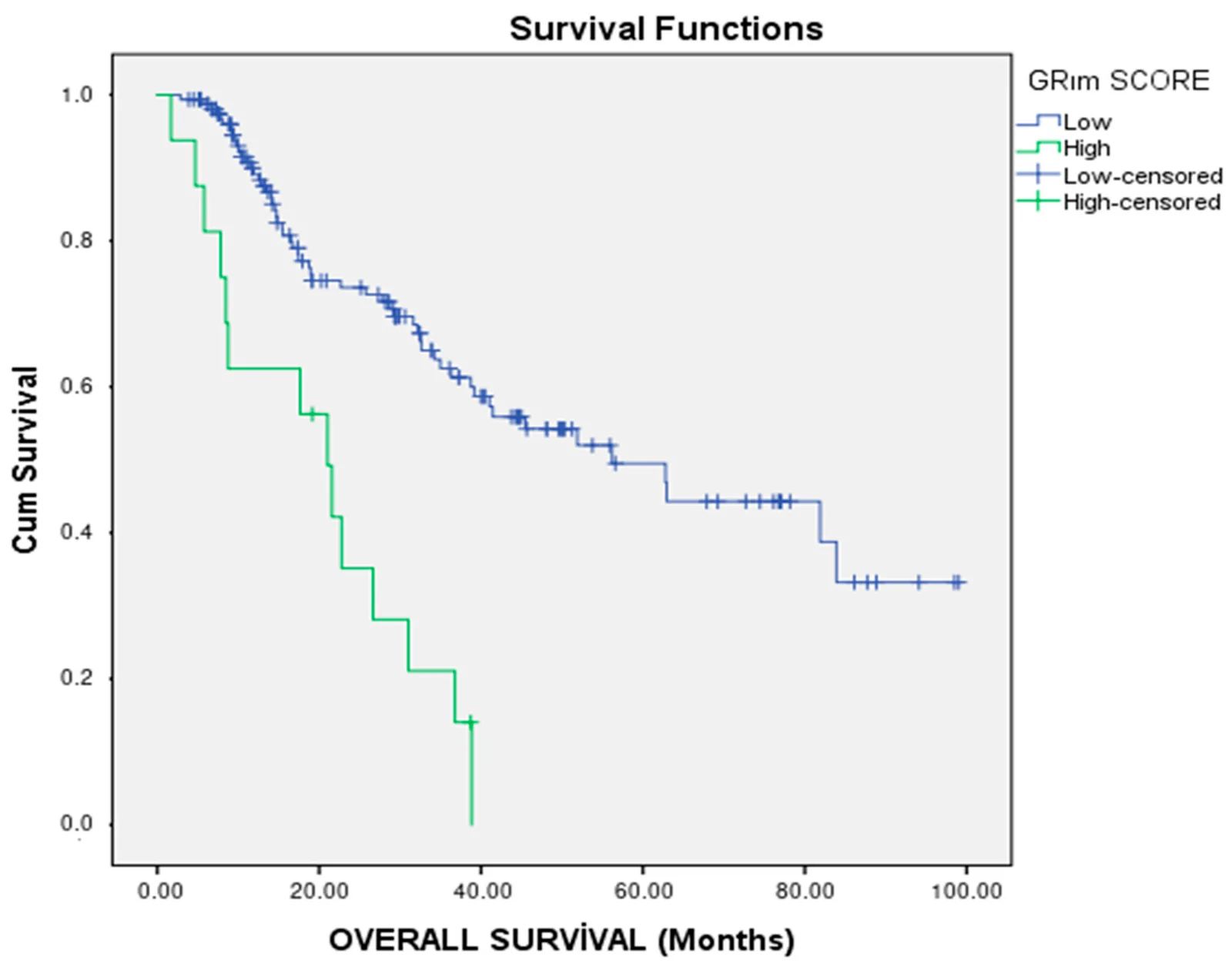
Kaplan–Meier curves for OS according to GRIM score. Patients were classified as having a low (0–1) or high (2–3) GRIM score. Median OS was 56.1 months (95% CI, 35.4–76.9) in the low GRIM score group and 21.0 months (95% CI, 14.1–28.0) in the high GRIM score group. Survival differed significantly between groups by the log-rank test (*p* < 0.001). Censored observations are indicated by tick marks. GRIM, Gustave Roussy Immune; OS, overall survival.

A total of 157 patients were classified as having a low GRIM score, whereas 16 patients had a high GRIM score. Median OS was 56.1 months (95% CI, 35.4–76.9) in the low GRIM score group and 21.0 months (95% CI, 14.1–28.0) in the high GRIM score group (log-rank *p* < 0.001). The 2-year OS rates were 73.6% and 62.1% in the low and high GRIM score groups, respectively. The corresponding 5-year OS rates were 49.5% and 0%. Kaplan–Meier survival curves according to the GRIM score category are shown in Figure 2.

### 3.5. Cox Regression Analysis for Overall Survival

In univariable Cox regression analysis, age ≥ 65 years, ypT3–4 disease, ypN-positive disease, aCCI ≥ 5, elevated CEA, high GRIM score, and receipt of adjuvant chemotherapy were associated with OS. In the multivariable Cox regression model, ypN-positive disease remained independently associated with worse OS (HR 2.65, 95% CI 1.14–6.19; *p* = 0.023). Similarly, aCCI ≥ 5 (HR 2.28, 95% CI 1.07–4.82; *p* = 0.031) and a high GRIM score (HR 2.44, 95% CI 1.11–5.36; *p* = 0.026) retained independent associations with inferior OS. Elevated CEA demonstrated a trend toward poorer survival but did not reach statistical significance in the multivariable model (HR 1.78, 95% CI 0.92–3.46; *p* = 0.086). Receipt of adjuvant chemotherapy was independently associated with improved OS (HR 0.29, 95% CI 0.14–0.60; *p* = 0.001). Age and pathological T stage were not independently associated with OS after adjustment.

The results of the univariable and multivariable Cox regression analyses are presented in Table 3.

### 3.6. Modified FLOT Prognostic Score

**Figure 3:**
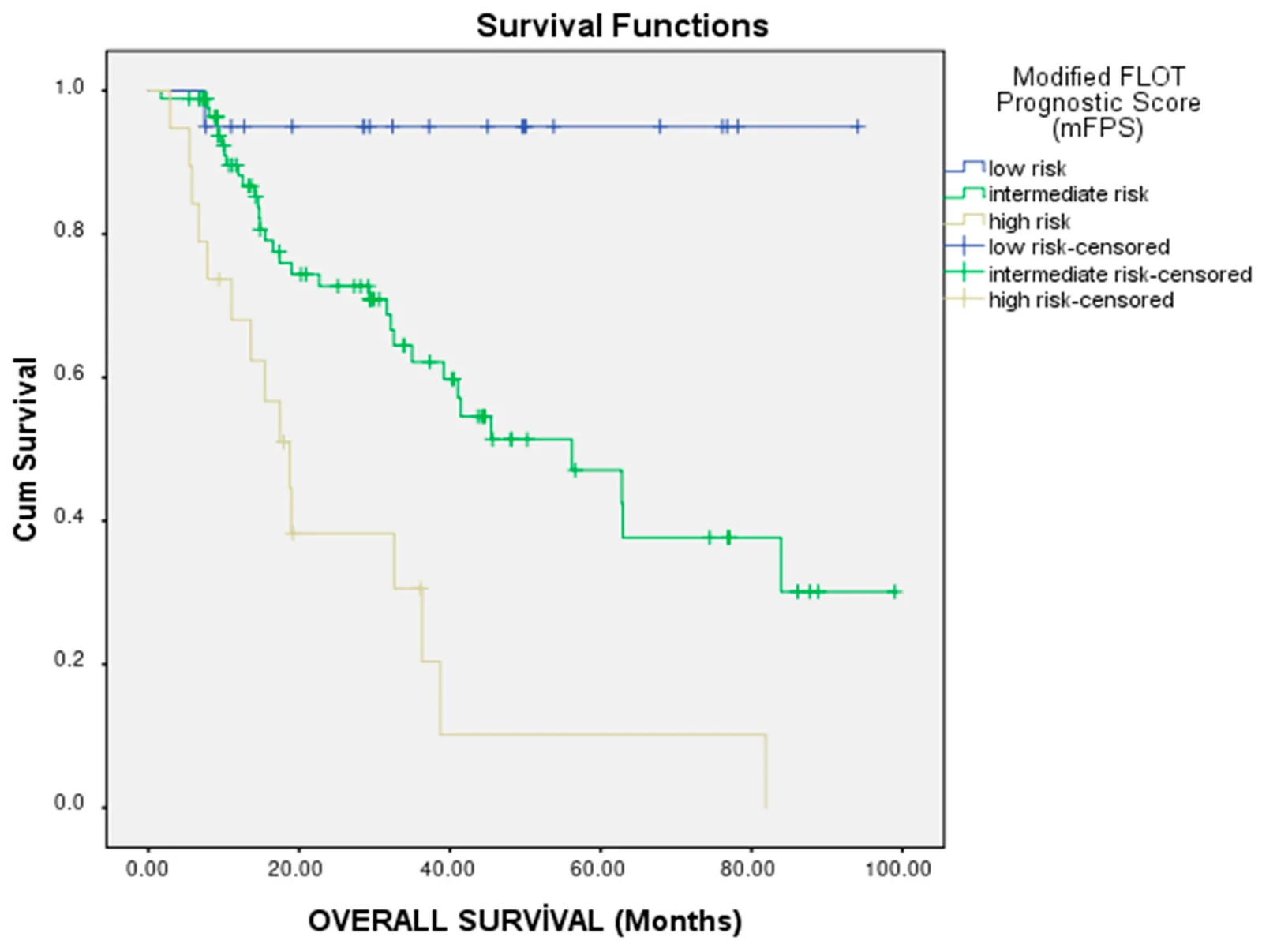
Overall survival according to modified FLOT Prognostic Score risk groups.

Among the 126 patients with complete data for all score components, 20 patients were classified as low risk, 87 as intermediate risk, and 19 as high risk. During follow-up, 1 death occurred in the low-risk group, compared with 32 deaths in the intermediate-risk group and 15 deaths in the high-risk group. Median OS was not reached in the low-risk group. Median OS was 56.1 months (95% CI, 33.7–78.6) in the intermediate-risk group and 18.7 months (95% CI, 12.3–25.1) in the high-risk group (log-rank *p* < 0.001). In univariable analysis, a stepwise worsening in OS was observed across mFPS risk groups. Compared with the low-risk group, the intermediate-risk group was associated with poorer OS (HR 10.3, 95% CI 1.4–75.6; *p* = 0.020), while the high-risk group had a substantially increased risk of death (HR 31.7, 95% CI 4.1–241.9; *p* = 0.001). In a multivariable model including age, pathological T stage, adjuvant treatment, and mFPS, the score remained independently associated with OS. Compared with the low-risk group, the intermediate-risk group remained associated with inferior OS (HR 8.97, 95% CI 1.22–65.9; *p* = 0.030), whereas the high-risk group had the poorest outcomes (HR 27.4, 95% CI 3.5–210.5; *p* = 0.001). Receipt of adjuvant chemotherapy was independently associated with improved OS (HR 0.35, 95% CI 0.19–0.66; *p* = 0.001). Kaplan–Meier survival curves according to mFPS risk groups are presented in Figure 3. Because the score was developed and evaluated within the same cohort, the observed findings should be considered exploratory and require external validation before clinical application.

Kaplan–Meier curves show OS according to modified FLOT Prognostic Score risk groups. Patients were stratified as low risk, intermediate risk, or high risk according to the cumulative score. Median OS was not reached in the low-risk group, compared with 56.1 months (95% CI, 33.7–78.6) in the intermediate-risk group and 18.7 months (95% CI, 12.3–25.1) in the high-risk group. Survival differed significantly across risk groups by log-rank test (*p* < 0.001). Censored observations are indicated by tick marks.

FLOT, fluorouracil, leucovorin, oxaliplatin, and docetaxel; mFPS, modified FLOT Prognostic Score; OS, overall survival.

## 4. Discussion

In this multicenter cohort of patients with gastric or gastroesophageal junction adenocarcinoma treated with perioperative FLOT chemotherapy, we evaluated the prognostic role of baseline inflammatory status, pretreatment CEA, postoperative nodal status, and comorbidity burden. At a median follow-up of 39.7 months, median OS was 41.4 months in the overall cohort. The main finding of this study was that survival after perioperative FLOT was not determined by residual disease burden alone. High GRIM score, ypN-positive disease, and higher comorbidity burden were independently associated with inferior survival. Furthermore, integrating these routinely available variables into the modified FLOT Prognostic Score enabled stratification of patients into distinct risk groups. Importantly, because the mFPS incorporates postoperative ypN status, it cannot be calculated before surgery and should not be interpreted as a tool for guiding initial treatment selection or the surgical approach. Rather, the mFPS should be considered an exploratory postoperative prognostic framework that integrates pretreatment host- and tumor-related characteristics with postoperative pathological nodal status. Accordingly, its potential clinical application is limited to postoperative risk stratification rather than pretreatment therapeutic decision-making. Median OS was not reached in the low-risk group, compared with 56.1 months in the intermediate-risk group and 18.7 months in the high-risk group.

Perioperative chemotherapy became an established strategy after the MAGIC trial showed an improvement in 5-year OS compared with surgery alone (36% vs. 23%), and FLOT4 later demonstrated longer median OS with FLOT than with epirubicin-, cisplatin-, and fluoropyrimidine-based regimens (ECF/ECX; 50 vs. 35 months), establishing FLOT as a preferred perioperative regimen for medically fit patients [3,24]. The survival outcomes observed in our cohort should be interpreted in the context of contemporary FLOT-based treatment and the heterogeneity of real-world patient populations. Unlike clinical trials, real-world cohorts include patients with variable comorbidity burden, postoperative recovery, treatment tolerance, and ability to complete adjuvant therapy. In our cohort, median OS was 41.4 months, the 5-year OS rate was 44%, and less than half of the patients completed planned adjuvant chemotherapy. These findings support the need for additional postoperative risk stratification beyond treatment allocation alone. The predominance of clinical stage III disease in our cohort is consistent with previously reported contemporary perioperative FLOT series, suggesting that the clinical characteristics of our study population are comparable to those observed in routine clinical practice [25].

More recently, perioperative treatment has moved beyond chemotherapy alone, with trials evaluating the addition of immune checkpoint inhibitors to FLOT-based or platinum–fluoropyrimidine-based regimens. These studies have improved pathological response and, in some settings, event-free survival; however, they also highlight the same clinical problem: outcomes remain heterogeneous even with intensified perioperative treatment [4,25,26]. Therefore, identifying patients who remain at high risk despite contemporary perioperative treatment is likely to become even more relevant as treatment options expand. In this context, postoperative pathological findings remain central, but they do not fully account for the variability in outcomes observed after treatment.

In the present cohort, residual pathological disease was common after neoadjuvant FLOT, and ypN-positive disease remained one of the strongest adverse prognostic factors in multivariable analysis (HR, 2.65; 95% CI, 1.14–6.19; *p* = 0.023). This finding is consistent with previous studies showing that post-treatment pathological stage, particularly nodal status, is strongly associated with survival in gastric cancer [6,27]. However, regression of the primary tumor does not fully capture the effect of treatment. Several studies have suggested that post-treatment nodal status is one of the most informative markers after neoadjuvant chemotherapy [7,28,29]. In the MAGIC pathology analysis, tumor regression was assessed using the Mandard tumor regression grading system; although poor regression and lymph node metastases were both associated with survival in univariate analysis, only lymph node status remained independently predictive of OS in multivariable analysis (HR, 3.36; 95% CI, 1.70–6.63; *p* < 0.001) [29]. Fujitani et al. similarly reported that posttherapy nodal status, rather than graded histologic response of the primary tumor, independently predicted survival after neoadjuvant chemotherapy [28]. Our findings are consistent with these observations and support the rationale for including ypN status as the postoperative treatment-response component of the mFPS.

The clinical meaning of ypN-positive disease after neoadjuvant therapy is therefore broader than anatomical staging. Persistent nodal involvement may indicate residual disease burden and incomplete sensitivity to chemotherapy, as suggested by previous analyses of post-treatment nodal status [7,28,29,30]. However, ypN status alone does not fully capture patient-related factors that may influence outcome. It does not account for baseline inflammation, nutritional reserve, comorbidity burden, or the ability to tolerate postoperative treatment. Consequently, patients with similar postoperative nodal stages may experience markedly different clinical courses. Some recover well and tolerate further treatment, whereas others remain frail, inflamed, or unable to complete adjuvant therapy. For this reason, postoperative risk assessment after perioperative FLOT may benefit from integrating pathological findings with host-related factors.

Systemic inflammation and nutritional status represent another part of this risk profile. Inflammation-based and nutrition-related indices such as NLR, GPS, PNI, systemic immune–inflammation index (SII), and other systemic inflammation-related scores have been associated with outcomes in gastric cancer and operable gastrointestinal malignancies [11,12,31,32,33,34,35]. In two meta-analyses, elevated NLR was associated with poorer survival in gastric cancer, with pooled HRs for OS of 1.83 and 1.98, respectively [31,32]. Low PNI has also been associated with worse OS and a higher risk of postoperative complications [33]. More recently, high pretreatment SII was associated with poorer overall and recurrence-free survival in gastric cancer in a meta-analysis of 30 studies [35]. Together, these markers provide prognostic information that is not fully reflected by TNM stage. This may be particularly relevant in patients receiving perioperative FLOT, as nutritional deterioration during FLOT has been associated with shorter recurrence-free survival [12].

In this context, the GRIM score offers a practical way to combine inflammatory and nutritional information with LDH. Although it was originally developed in patients treated in immunotherapy phase I trials, subsequent data suggest that its prognostic value is not limited to that setting [9,13]. In a meta-analysis including 15 studies and 4997 patients, a high GRIM score was associated with poorer OS and progression-free survival across different cancer types [9]. Together, albumin, LDH, and NLR offer a practical summary of nutritional status, inflammatory burden, and disease aggressiveness. In our cohort, high GRIM score remained associated with shorter OS in multivariable analysis (HR, 2.44; 95% CI, 1.11–5.36; *p* = 0.026). This does not establish GRIM as a treatment-selection tool in this setting. Rather, it appears to identify patients with a less favorable baseline host-tumor profile before perioperative treatment.

Comorbidity burden also remained clinically relevant in our cohort. aCCI was available in 136 patients, with a median value of 4, and aCCI ≥ 5 was independently associated with shorter OS (HR, 2.28; 95% CI, 1.07–4.82; *p* = 0.031). The aCCI was developed to classify comorbid conditions associated with mortality risk and is widely used in clinical research [14,20]. In perioperative gastric cancer, comorbidities may influence outcomes through reduced tolerance to chemotherapy, increased postoperative morbidity, delayed recovery, lower probability of completing adjuvant therapy, and competing causes of death. These observations highlight that outcomes after perioperative FLOT are influenced not only by tumor-related factors but also by the patient’s baseline physiological reserve and ability to tolerate multimodal treatment.

The rationale for the mFPS was to integrate prognostic information that is often considered separately in routine practice. The score combines systemic inflammation and nutritional status, tumor marker elevation, residual nodal disease, and comorbidity burden, each representing a distinct component of postoperative risk. In the present study, the mFPS stratified patients into groups with markedly different outcomes, with median OS not reached in the low-risk group, compared with 56.1 months in the intermediate-risk group and 18.7 months in the high-risk group. These findings suggest that combining host-related and tumor-related factors may provide a more comprehensive assessment of risk after perioperative FLOT than any single variable alone. However, this observation should not be overstated. The mFPS was developed and evaluated within the same retrospective cohort and was not externally validated. It should therefore be considered an exploratory risk stratification approach rather than a validated clinical prediction tool.

Several prognostic models incorporating clinicopathological characteristics, inflammatory indices, nutritional markers, or molecular features have been proposed for gastric cancer [36]. Recent reviews have highlighted that prognostic assessment is increasingly moving beyond conventional pathological staging toward multidimensional approaches integrating tumor biology and host-related characteristics [5,36]. Within this context, the mFPS was developed to combine complementary prognostic information derived from postoperative pathological findings, systemic inflammation and nutritional status, tumor marker burden, and comorbidity using routinely available clinical variables. Unlike models that primarily focus on a single prognostic domain, the mFPS was designed to integrate multiple clinically relevant prognostic domains into a simple postoperative risk stratification framework. Nevertheless, the mFPS remains exploratory and requires independent validation before its potential role in routine clinical practice can be established.

The decision to assign equal weight to each component was made a priori, based on the conceptual framework of the score, rather than according to the magnitude of the regression coefficients observed in the derivation cohort. Although coefficient-based weighting may improve model performance within a single dataset, it may also reduce reproducibility when applied to an independent population, particularly in retrospective studies with a limited complete-case sample. Furthermore, deriving regression coefficient-based weights and comparing weighted and unweighted versions of the score within the same derivation cohort would itself increase the risk of overfitting and could lead to overly optimistic estimates of predictive performance. For this reason, we favored a simpler approach in which each prognostic domain contributed equally to the final score. Whether alternative weighting strategies improve predictive performance should be evaluated in future studies using appropriate internal validation procedures (e.g., bootstrap resampling) and independent external validation, including discrimination measures such as Harrell’s C-index.

The derivation and evaluation of the mFPS within the same retrospective cohort represents an important methodological limitation. Because neither internal nor external validation was performed, the prognostic performance observed in the present study may not fully reflect the performance of the score in other patient populations, and the possibility of overfitting cannot be excluded. Moreover, no formal internal validation procedures, such as bootstrap resampling or cross-validation, were performed, and no suitable external validation cohort was available. These findings should therefore be interpreted with appropriate caution until the score has been evaluated in independent cohorts. Future studies should assess its performance using both internal and external validation strategies and determine whether its prognostic value is maintained across different clinical settings. In addition, no internal validation procedures, such as bootstrap resampling, were performed during score development. Furthermore, the relatively wide confidence intervals observed in the low-risk subgroup likely reflect the limited number of events and should be considered when interpreting the prognostic performance of the proposed score. Finally, independent validation in external cohorts, ideally through prospective studies, is required before the mFPS can be considered for routine clinical implementation.

The timing of the mFPS should also be considered. Because ypN status is one of its components, the score is not purely a pretreatment model. Rather, it is best viewed as a postoperative risk stratification tool. Its potential value may lie in identifying patients who remain at increased risk despite completion of multimodal treatment. Whether such patients could benefit from alternative surveillance strategies, supportive interventions, or enrollment in clinical trials requires prospective evaluation and should be considered hypothesis-generating.

The association between adjuvant treatment and longer OS also requires cautious interpretation. In retrospective perioperative cohorts, postoperative chemotherapy is not randomly assigned. Patients who receive adjuvant therapy are usually those who recover adequately from surgery, do not experience early disease progression, and remain fit enough to continue treatment. Therefore, the observed association between adjuvant treatment and survival may reflect treatment fitness and selection bias rather than a direct treatment effect. This issue is well recognized in perioperative gastric cancer, where postoperative treatment completion is often lower than preoperative treatment completion in both trials and real-world series [3,24,37]. In a recent real-world FLOT cohort, adjuvant chemotherapy was initiated in only 42.9% of patients, and its apparent benefit varied according to histopathologic response, with the strongest signal observed among patients with limited tumor regression [37]. For this reason, the association between adjuvant chemotherapy and survival in the present study should be interpreted as observational rather than causal.

This study has several strengths. It included patients treated across seven oncology centers and therefore reflects routine clinical practice rather than a highly selected trial population. Patients were included if FLOT was initiated with curative intent, even if they did not ultimately undergo surgery because of progression, death, or refusal. This design allowed the analysis to reflect the real-world perioperative treatment pathway, including patients who did not proceed to surgery after initiating neoadjuvant therapy. Another strength is that the proposed score is based on variables that are biologically plausible, inexpensive, and available in most oncology centers.

The limitations should also be acknowledged. The retrospective design introduces the possibility of selection bias, missing data, and residual confounding. In addition, the mFPS was developed and assessed within the same cohort, which may overestimate its prognostic performance. Current guidance for prognostic model reporting emphasizes internal validation, calibration assessment, and external validation before clinical implementation [17,38]. Another limitation of the present study is the use of complete-case analysis for the development of the mFPS. Because all four predefined variables were considered essential for score calculation, only patients with complete information for GRIM score, CEA, ypN status, and aCCI were included in the score analysis. Consequently, the exclusion of patients with missing data reduced the effective sample size and may have introduced selection bias, which should be considered when interpreting the performance of the proposed score. Comparison of baseline characteristics between patients included in and excluded from the complete-case analysis demonstrated generally similar distributions across most variables; however, excluded patients had poorer ECOG PS (Supplementary Table S2), indicating that some degree of selection bias cannot be completely excluded. Given the retrospective design and the exploratory nature of the study, we considered complete-case analysis preferable to imputing missing values for the score components, as imputation would have introduced additional assumptions into the derivation of the score. Therefore, the proposed score should be interpreted as hypothesis-generating and requires independent validation in larger prospective cohorts with more complete datasets.

MMR status was also unavailable in a substantial proportion of patients and was not incorporated into the model, although molecular features may influence prognosis and treatment response in gastric cancer [1,4,5,26]. Finally, surgical approach, pathological assessment, supportive care, and follow-up practices may have varied across centers, and the number of patients in some subgroups, particularly the high GRIM and high mFPS groups, was limited. Long-term survival estimates in these groups should therefore be interpreted with caution.

## 5. Conclusions

In conclusion, this multicenter retrospective study showed that survival after perioperative FLOT was influenced not only by residual disease burden but also by host-related factors. A high GRIM score, ypN-positive disease, and higher comorbidity burden were independently associated with inferior OS. The mFPS integrated these routinely available variables and identified patient groups with markedly different outcomes. However, because the score was developed and evaluated within the same retrospective cohort, it should be regarded as an exploratory risk stratification framework requiring external validation before clinical application.

## Figures and Tables

**Table 1:** Baseline patient and disease characteristics.

Variables		*n* = 173 (%)
Gender	Female	46 (26.6)
	Male	127 (73.4)
Age (years)	Median (IQR)	61 (54.5–67)
	≥65	57 (32.9)
ECOG PS	PS 0	102 (59)
	PS 1	65 (37.6)
	PS ≥ 2	6 (3.5)
Clinical stage	1	7 (4)
	2	43 (24.9)
	≥3	123 (71.1)
Clinical T stage	1	7 (4)
	2	53 (30.6)
	3	89 (51.4)
	4	24 (13.9)
Clinical N stage	0	26 (15)
	1	65 (37.6)
	2	59 (34.1)
	3	23 (13.3)
Tumor localization	Cardia	59 (33.5)
	Corpus–fundus	49 (28.3)
	Antrum	48 (27.7)
	Gastroesophageal junction	15 (8.7)
	Linitis plastica	3 (1.7)
Histopathologic subtype	Adenocarcinoma	129 (74.6)
	Signet ring cell carcinoma	40 (23.1)
	Other	4 (2.3)
MMR status, *n* (%)	pMMR	90 (52)
	dMMR	9 (5.2)
	Unknown	74 (42.8)
CEA	Median (IQR)	2.81 (1.49–7.11)
CA 19-9	Median (IQR)	17 (7.1–31.61)
PET Primary Tumor SUVmax	Median (IQR)	10.6 (6.47–16.55)
aCCI (*n* = 136)	Median (IQR)	4 (3–5)
	≥5	68 (50)
GRIM Score	Low (0–1)	157 (91.9)
	High (2–3)	16 (9.2)
NLR	Median (IQR)	2.5 (0.95–16.1)

Data are presented as *n* (%) unless otherwise indicated. Continuous variables are reported as median (interquartile range). Percentages were calculated using the total study population as the denominator unless otherwise specified. MMR status was available for 99 patients. Age-Adjusted Charlson Comorbidity Index was available for 136 patients. GRIM score was calculated before treatment initiation using serum albumin, LDH, and NLR. CA 19-9, carbohydrate antigen 19-9; CEA, carcinoembryonic antigen; aCCI, Age-Adjusted Charlson Comorbidity Index; ECOG PS, Eastern Cooperative Oncology Group performance status; GRIM, Gustave Roussy Immune; IQR, interquartile range; LDH, lactate dehydrogenase; MMR, mismatch repair; NLR, neutrophil-to-lymphocyte ratio; pMMR, proficient mismatch repair; dMMR, deficient mismatch repair; SUVmax, maximum standardized uptake value.

**Table 2:** Treatment Characteristics and Outcomes.

		*n* (%)
Neoadjuvant FLOT regimen cycles	Median (IQR)	4 (4–4)
Completion rate of neoadjuvant chemotherapy		142 (82.1)
Patients undergoing surgery		164 (94.8)
Reason for not undergoing surgery	Disease progression	6 (3.5)
	Death	1 (0.6)
	Patient refusal	2 (1.2)
Pathologic complete response		7 (4.0)
Pathologic T stage	ypT0	8 (4.6)
	ypT1	20 (11.6)
	ypT2	14 (8.1)
	ypT3	74 (42.8)
	ypT4	33 (19.1)
	Unknown	24 (13.9)
Pathologic N stage	N0	55 (31.8)
	N1	29 (16.8)
	N2	30 (17.3)
	N3	35 (20.3)
	Unknown	24 (13.9)
Adjuvant treatment		133 (76.9)
Adjuvant FLOT regimen cycles		4 (1–4)
Completion rate of adjuvant chemotherapy		83 (47.9)
Reason for non-completion of adjuvant therapy	Toxicity	31 (17.9)
	Disease progression	12 (6.9)
	Patient refusal	16 (9.2)
	Death	5 (2.9)
	Other	2 (1.2)
Progression		61 (35.3)
Exitus		69 (39.9)

Data are presented as *n* (%) unless otherwise indicated. Continuous variables are reported as median (interquartile range). Pathological T and N categories were evaluated among patients who underwent surgery and had available postoperative pathological staging. Patients who did not undergo surgery or had unavailable pathological staging were categorized as unknown for ypT and ypN status. Reasons for non-completion of adjuvant chemotherapy were recorded among patients who did not complete planned postoperative treatment. FLOT, fluorouracil, leucovorin, oxaliplatin, and docetaxel; IQR, interquartile range; ypN, post-treatment pathological nodal stage; ypT, post-treatment pathological tumor stage.

**Table 3:** Cox regression analysis for overall survival.

Variables		Univariate Analysis		Multivariate Analysis	
		HR (95% CI)	*p*	HR (95% CI)	*p*
Gender	Female	1.3 (0.76–2.35)	0.30		
	Male				
Age	≥65 years	1.69 (1.04–2.75)	0.03	0.62 (0.28–1.38)	0.39
	<65 years				
ECOG PS	PS ≥ 1	1.22 (0.76–1.97)	0.40		
	PS 0				
Histology	Adenocarcinoma	0.86 (0.48–1.53)	0.61		
	Signet ring cell carcinoma				
Pathologic T Stage	ypT3-4	2.8 (1.34–6.02)	0.006	1.58 (0.62–4.04)	0.33
	≤ypT2				
Pathologic N Stage	ypN1-3	1.5 (1.10–2.16)	0.01	2.65 (1.14–6.19)	0.023
	ypN0				
Adjuvant Treatment	Yes	0.36 (0.21–0.60)	0.001	0.29 (0.14–0.60)	0.001
	No				
aCCI	≥5	2.6 (1.47–4.57)	0.01	2.28 (1.07–4.82)	0.031
	<5				
CEA	≥5	2.40 (1.42–4.07)	0.001	1.78 (0.92–3.46)	0.086
	<5				
CA 19-9	<33	1.35 (0.76–2.42)	0.32		
	≥33				
GRIM Score	High (2–3)	1.95 (1.44–2.63)	0.001	2.44 (1.11–5.36)	0.026
	Low (0–1)				

Hazard ratios were estimated using Cox proportional hazards regression. Variables were entered into the multivariable model based on clinical relevance and/or association with overall survival in univariate analysis. Missing data were not imputed, and analyses were performed using available cases for each variable. Adjuvant treatment was included as a treatment-delivery variable and should be interpreted cautiously because receipt of postoperative therapy may be influenced by postoperative recovery, early progression, toxicity, and treatment fitness. CA 19-9, carbohydrate antigen 19-9; CEA, carcinoembryonic antigen; aCCI, Age-Adjusted Charlson Comorbidity Index; CI, confidence interval; ECOG PS, Eastern Cooperative Oncology Group performance status; GRIM, Gustave Roussy Immune; HR, hazard ratio; ypN, post-treatment pathological nodal stage; ypT, post-treatment pathological tumor stage.

## Data Availability

The data presented in this study are available on reasonable request from the corresponding author. The data are not publicly available due to ethical and privacy restrictions.

## Supplementary Materials

The following supporting information can be downloaded at https://www.mdpi.com/article/10.3390/jcm15166300/s1. Table S1: Components and risk categories of the modified FLOT Prognostic Score; Table S2: Comparison of baseline characteristics between patients included in and excluded from the complete-case mFPS analysis.

## Abbreviations

The following abbreviations are used in this manuscript:

aCCI	Age-Adjusted Charlson Comorbidity Index
CA 19-9	Carbohydrate antigen 19-9
CEA	Carcinoembryonic antigen
CI	Confidence interval
dMMR	Deficient mismatch repair
ECOG PS	Eastern Cooperative Oncology Group performance status
FLOT	Fluorouracil, leucovorin, oxaliplatin, and docetaxel
GEJ	Gastroesophageal junction
GPS	Glasgow Prognostic Score
GRIM	Gustave Roussy Immune
HR	Hazard ratio
IQR	Interquartile range
LDH	Lactate dehydrogenase
mFPS	Modified FLOT Prognostic Score
MMR	Mismatch repair
NLR	Neutrophil-to-lymphocyte ratio
OS	Overall survival
pCR	Pathological complete response
PET/CT	Positron emission tomography/computed tomography
pMMR	Proficient mismatch repair
PNI	Prognostic Nutritional Index
SSI	Systemic Immune–Inflammation Index
SUVmax	Maximum standardized uptake value
ypN	Post-treatment pathological nodal stage
ypT	Post-treatment pathological tumor stage
