# Supplementary material for: Integrated Prognostic Stratification After Perioperative FLOT in Gastric and Gastroesophageal Junction Adenocarcinoma: A Multicenter Real-World Study
Source: J Clin Med. 2026 Aug 14;15(16):6300. doi: 10.3390/jcm15166300 (PMC13513156; doi:10.3390/jcm15166300)
Supplement: Supplementary file 1 [file jcm-15-06300-s001.zip › jcm-4452233-supplementary.pdf]

Supplementary Table S1. Components and risk categories of the modified FLOT Prognostic Score

Component	Adverse factor	Points
GRIM score	High GRIM score (2–3)	1
CEA	≥ 5 ng/mL	1
ypN status	ypN-positive disease	1
Age-adjusted Charlson Comorbidity Index	aCCI ≥ 5	1
**Total score**	**Range 0–4**	
Low risk	0 points	
Intermediate risk	1–2 points	
High risk	3–4 points	

The mFPS was calculated only in patients with complete data for all four score components: pretreatment GRIM score, pretreatment CEA level, postoperative ypN status, and Charlson Comorbidity Index. One point was assigned for each adverse factor. Patients with missing data for any score component were excluded from mFPS-specific analyses but remained in the overall survival cohort when follow-up data were available.

CEA, carcinoembryonic antigen; aCCI, Age-adjusted Charlson Comorbidity Index; GRIM, Gustave Roussy Immune; mFPS, modified FLOT Prognostic Score; ypN, post-treatment pathological nodal stage.

Supplementary Table S2. Comparison of baseline characteristics between patients included in and excluded from the complete-case mFPS analysis.

Variable		Included in mFPS (n = 126)	Excluded because of missing data (n = 47)	p
Age	Median (IQR)	61 (55-66)	59 (55-66)	0.57
	≥65 years	43 (34.2)	14 (29.7)	0.58
Gender	Female	30 (23.8)	16 (34.1)	0.17
	Male	96 (76.2)	31 (65.9)	
ECOG PS	PS 0	82 (65.1)	20 (42.6)	0.007
	PS 1	42 (33.3)	23 (48.9)	
	PS ≥2	2 (1.6)	4 (8.5)	
Clinical Stage	1	6 (4.8)	1 (2.1)	0.76
	2	30 (23.8)	13 (27.7)	
	≥ 3	90 (71.4)	33 (70.2)	
Histopathologic subtype	Adenocarcinoma	90 (71.4)	39 (83)	0.28
	Signet ring cell carcinoma	33 (26.2)	7 (14.9)	
	Other	3 (2.4)	1 (2.1)	
Adjuvant Treatment		99 (78.5)	34 (72.3)	0.71
Recurrence		41 (32.6)	20 (42.5)	0.34
Death		48 (38.0)	21 (44.6)	0.43

Continuous variables were compared using the Mann–Whitney U test. Categorical variables were compared using the χ^2 test or Fisher's exact test, as appropriate.
